# Emerging Manifestations of Cesarean Scar Defect in the Reproductive-Age Group

**DOI:** 10.7759/cureus.97287

**Published:** 2025-11-19

**Authors:** Shahida Sultan, Khawaja Fawad Parvez, Mohammad Shayan Khan

**Affiliations:** 1 Obstetrics and Gynecology, Lady Reading Hospital, Peshawar, PAK; 2 Obstetrics and Gynecology, Pak International Medical College, Peshawar, PAK

**Keywords:** abnormal uterine bleeding, cesarean scar defect, isthmocele, pelvic pain, secondary infertility, transvaginal sonography

## Abstract

Introduction: Cesarean scar defect (CSD), also referred to as isthmocele or niche, has emerged as a significant gynecological issue among reproductive-age women, attributed to rising cesarean delivery rates and improved imaging modalities. It often manifests as abnormal uterine bleeding (AUB), pelvic pain, and infertility. This study aimed to assess the clinical profile, imaging characteristics, and reproductive associations of CSD in women of reproductive age.

Materials and methods: This cross-sectional study was conducted in the Department of Obstetrics and Gynecology from July 2022 to December 2023. Women aged 20-45 years with a history of cesarean delivery presenting with menstrual irregularities, pelvic pain, or infertility were evaluated using transvaginal sonography (TVS; GE Voluson E8, GE Healthcare, Chicago, IL, USA). Cases suspected of CSD underwent confirmation with saline infusion sonohysterography (GE Healthcare, Chicago, IL, USA) or hysteroscopy (Karl Storz GmbH & Co. KG, Tuttlingen, Germany). Clinical features, imaging findings, and reproductive outcomes were analyzed using SPSS Statistics version 24.0 (IBM Corp. Released 2016. IBM SPSS Statistics for Windows, Version 24.0. Armonk, NY: IBM Corp.), with p ≤ 0.05 considered statistically significant.

Results: A total of 100 women were included, with a mean age of 31.6 ± 4.2 years. AUB was the most common symptom, 78 (78%), followed by pelvic pain, 46 (46%), and secondary infertility, 39 (39%). TVS identified CSD in 89 (89%) of cases; it was confirmed hysteroscopically in 81 (81%). Women with ≥2 previous cesarean sections had significantly deeper defects (p = 0.02). A niche depth >3 mm, as defined in prior literature, was significantly associated with secondary infertility (p = 0.01). Emergency cesarean delivery correlated with irregular niche formation (p = 0.03).

Conclusions: CSD is an underdiagnosed but clinically important condition contributing to gynecological morbidity and infertility. Early recognition through imaging and heightened clinical awareness can improve reproductive outcomes and prevent complications in subsequent pregnancies.

## Introduction

Cesarean section (CS) is one of the most commonly performed obstetric surgeries worldwide, and its global prevalence has been steadily increasing. The estimated overall CS rate is approximately 21.1% of all deliveries, representing a marked increase over recent decades [1]. While the World Health Organization has historically recommended a CS rate of approximately 10-15% [2], many regions now exceed this value, raising concerns about the long-term sequelae of uterine surgery. An often under-recognized complication of lower-segment cesarean delivery is the development of a CS defect (CSD), also known as an isthmocele, uterine niche, or scar pouch [3]. The defect manifests as focal thinning or indentation of the myometrium at the previous uterine incision site and is frequently detectable by transvaginal ultrasound, saline infusion sonohysterography, or hysteroscopy [4].

The prevalence of CSD among women with prior CS varies widely in the literature, mainly due to differences in study populations and diagnostic modalities. In asymptomatic women, detection rates are typically lower, ranging from 24% to 32% with 2D transvaginal sonography (TVS) [5], whereas 3D ultrasound or saline-infusion sonohysterography identifies defects in 56-70% of cases [6]. The estimates of pooled prevalence in symptomatic populations range from approximately 43% to 46% [7]. Other prospective cohorts have reported that as many as 42.5% of women develop cesarean scar abnormalities within three years of their first CS [8]. However, not all niches are symptomatic; only a subset of women with CSD present with clinical complaints. Clinically, CSD may present as a heterogeneous spectrum of signs and symptoms. The most frequently reported complaint is abnormal uterine bleeding (AUB), mainly persistent spotting or post-menstrual bleeding lasting beyond the usual menstrual period [9]. A recent meta-analysis combining 10 studies (n = 1,183 women with CSD) estimated an AUB prevalence of approximately 46% (95% CI 27-64%) and found that women with CSD had over a 3.2-fold increased risk of AUB compared with women without defects (RR = 3.22, 95% CI 1.83-5.66) [10]. Other symptoms include pelvic pain, dysmenorrhea, dyspareunia, and secondary infertility. In a systematic review focusing on niche and pelvic pain, prevalence rates of dysmenorrhea (~38.2%), dyspareunia (~28.2%), and chronic pelvic pain (~26.8%) were reported among women with confirmed niches [11].

The pathophysiology underlying the development of CSD symptoms is multifactorial. Proposed mechanisms include retention of menstrual blood within the defect (impaired drainage), altered local hemodynamics due to neovascularization, local inflammation, fibrosis [12], and impaired contractility of the residual myometrium at the scar site [13]. Additionally, several surgical, anatomical, and patient factors have been implicated in niche formation and its severity, such as low incision placement, single-layer versus double-layer closure, uterine retroflexion [14], multiple CSs, suboptimal wound healing, infection, and anemia [15].

Importantly, CSD is not solely a gynecologic concern; it has been associated with adverse reproductive outcomes in subsequent pregnancies, including cesarean scar ectopic pregnancies, placenta accreta spectrum disorders [16], and the risk of uterine rupture [17]. Moreover, the presence of a niche may complicate intrauterine procedures such as IUD insertion, embryo transfer, and uterine evacuation [7].

Despite growing awareness, there remains a gap in comprehensive clinical data, particularly from low- and middle-income countries such as Pakistan, regarding the presentation, diagnostic patterns, and reproductive implications of CSD. Most available evidence originates from high-income settings where standardized imaging protocols and advanced diagnostic modalities are routinely accessible. However, in lower-resource settings, such as ours, diagnostic evaluation often relies on conventional 2D ultrasound, leading to potential underdiagnosis and limited understanding of the condition’s clinical spectrum. This underscores the need for region-specific research to better characterize CSD in relation to symptomatology, imaging features, and reproductive outcomes. Therefore, this study aimed to investigate the clinical presentations, imaging correlates, and fertility-related associations of CSD among women of reproductive age in Pakistan.

## Materials and methods

Study design and setting

This cross-sectional observational study was conducted in the Department of Obstetrics and Gynecology over an 18-month period from July 2022 to December 2023. Women aged 20-45 years with a history of one or more cesarean deliveries and presenting with AUB, pelvic pain, or secondary infertility were evaluated for CSD. One hundred women who met the eligibility criteria were included consecutively from outpatient and radiology clinics. The study protocol was reviewed and approved by the Lady Reading Hospital Medical Teaching Institution Institutional Review Board (approval no. 221/LRH/MTI). Written informed consent was obtained from all participants before their inclusion in the study.

Eligibility criteria

Women between the ages of 20 and 45 years who had undergone one or more previous cesarean deliveries and presented with gynecological complaints, such as menstrual irregularities, pelvic pain, or infertility, were considered eligible for inclusion in the study. Only patients in whom a CSD was identified through TVS (GE Voluson E8, GE Healthcare, Chicago, IL, USA), saline infusion sonohysterography (GE Healthcare, Chicago, IL, USA), or hysteroscopy (Karl Storz GmbH & Co. KG, Tuttlingen, Germany) were enrolled. Participants were excluded if they had congenital uterine anomalies, such as a septate or bicornuate uterus, uterine or cervical malignancies, or active pelvic infections, as these factors could confound menstrual or reproductive outcomes unrelated to scar defects. Patients with incomplete clinical or imaging records were excluded from the final analysis to ensure data integrity and diagnostic accuracy.

Clinical and imaging assessment

All participants underwent a detailed clinical evaluation, including obstetric and gynecological history, number and indication of previous CS, and associated risk factors (e.g., emergency procedure, infection, and diabetes). Initial screening for CSD was performed using TVS with a high-frequency (7.5 MHz) probe. The presence of a niche was defined as a triangular or anechoic defect in the anterior uterine isthmus in sagittal view. The depth of the defect was measured from the base to the apex in millimeters. A defect depth greater than 3 mm was considered diagnostic of a clinically significant CSD, consistent with prior studies [18,19]. When TVS findings were equivocal, saline infusion sonohysterography or diagnostic hysteroscopy was performed to confirm the diagnosis. The presence of intrauterine fluid accumulation, residual myometrial thickness (RMT), and niche morphology was also recorded. Two independent gynecologists reviewed each imaging report to ensure diagnostic accuracy; discrepancies were resolved by consensus. Formal inter-observer agreement statistics were not computed due to the descriptive design.

Data collection

Demographic details, clinical presentation, imaging parameters, and reproductive histories were recorded using a standardized protocol. Variables analyzed included age, parity, number of previous CS, type of CS (elective vs. emergency), and the main presenting symptoms. The depth of CSD, presence of intrauterine fluid, and correlation with infertility were noted.

Statistical analysis

Data analysis was performed using SPSS Statistics version 24.0 (IBM Corp. Released 2016. IBM SPSS Statistics for Windows, Version 24.0. Armonk, NY: IBM Corp.). Continuous variables are expressed as mean ± standard deviation (SD), and categorical variables are presented as frequencies and percentages. The chi-square test and Student’s t-test were used to evaluate associations between categorical and continuous variables, respectively. Statistical significance was assessed at p ≤ 0.05 with a 95% confidence level.

## Results

A total of 100 women aged 20-45 years were included in the study, with a mean age of 31.6 ± 4.2 years. The majority of participants (72%) had undergone two or more previous CS. Table 1 summarizes the baseline demographic and clinical characteristics of the study cohort.

**Table 1:** Baseline demographic and clinical characteristics of study participants (n = 100) Values expressed as mean ± SD or percentage (%). AUB: abnormal uterine bleeding, SD: standard deviation

Variable	N (%)
≥2 previous C-sections	72 (72%)
AUB	78 (78%)
Pelvic pain	46 (46%)
Secondary infertility	39 (39%)

The most frequent clinical presentation was AUB, reported by 78 participants (78%). Other commonly observed symptoms include pelvic pain in 46 (46%) and secondary infertility in 39 (39%). These findings underscore the predominance of menstrual disturbances as the leading symptom in patients with CSD. TVS successfully detected CSD in 89 (89%) cases, which was subsequently confirmed by hysteroscopic evaluation in 81 patients (81%). The mean defect depth was 3.4 ± 1.1 mm (Table 2).

**Table 2:** Diagnostic imaging characteristics of CSD on TVS and hysteroscopy Values expressed as mean ± SD or percentage (%). CSD: cesarean scar defect, SD: standard deviation, TVS: transvaginal sonography

Imaging modality	N (%)/mean ± SD
TVS detection rate	89 (89%)
Hysteroscopic confirmation	81 (81%)
Average defect depth	3.4 ± 1.1 mm

Among 39 women with secondary infertility, intrauterine fluid accumulation within the scar defect was detected in 29 (74%), and 27 (69%) of these patients had niche depths exceeding 3 mm. In 14 (35%) cases, saline infusion sonohysterography was required to clarify the findings when the initial TVS results were inconclusive, reflecting the added diagnostic value of contrast imaging (Table 3).

**Table 3:** Subgroup analysis among infertile women with caesarean scar defect (n = 39) Data expressed as percentages (%).

Finding	N (%)
Infertile patients with intrauterine fluid	29 (74%)
Infertile patients with a defect >3 mm	27 (69%)
Patients needing sonohysterography	14 (35%)

A statistically significant association was observed between the number of prior cesarean deliveries and defect severity, with deeper niches observed in women with two or more cesarean deliveries (p = 0.02). Similarly, niche depth greater than 3 mm showed a strong correlation with secondary infertility (p = 0.01), suggesting a clinically meaningful relationship between scar morphology and reproductive outcomes. Furthermore, a significant association was noted between emergency cesarean delivery and irregular niche formation (p = 0.03), implying that suboptimal surgical conditions and healing dynamics may contribute to the development of defective scars (Table 4).

**Table 4:** Association of clinical and surgical factors with CSD characteristics p-values calculated using the chi-square test. p < 0.05 is considered significant. CS: cesarean section, CSD: cesarean scar defect

Variable compared	Association observed	p-value
≥2 previous CS vs. defect depth	Deeper defects in women with ≥2 CS	0.02
Niche depth >3 mm vs. secondary infertility	Increased infertility with deeper niches	0.01
Emergency CS vs. irregular niche formation	Irregular niches are more frequent after emergency CS	0.03

Collectively, these results highlight that CSD is prevalent among symptomatic women with prior cesarean deliveries. This condition not only manifests with gynecological morbidity, such as abnormal bleeding and pain, but also exerts measurable reproductive implications, particularly when the defect depth exceeds 3 mm.

## Discussion

In this study, we evaluated the clinical presentations, imaging characteristics, and reproductive ramifications of CSD in women of reproductive age. Our findings reinforce that CSD is not merely an incidental imaging finding but often correlates with significant gynecologic symptoms, especially AUB, pelvic pain, and secondary infertility, and that morphological parameters such as defect depth are clinically relevant. Our observed prevalence of AUB among symptomatic women with CSD (78%) aligns with meta-analyses showing that women with CSD are significantly more likely to report AUB compared to those without CSD [20]. A recent study reported a pooled prevalence of AUB of about 46% among women with CSD, rising to ~77% in populations selected for gynecologic imaging indications [10].

Furthermore, our data showing that deeper defect depths are associated with secondary infertility mirrors the results reported elsewhere. For example, a cross-sectional study found that reduced RMT, especially < 2.5 mm, was significantly associated with infertility and heavy menstrual bleeding [21]. In meta-analytic [22] and cohort [23] data, larger and deeper niches are repeatedly associated with more severe bleeding symptoms and poorer reproductive outcomes. We also observed that women with ≥2 prior CS tended to have more severe defect morphology. This concurs with the literature, which demonstrates that multiple CS, emergency CS, and specific surgical techniques or incision placements are risk factors for more pronounced niche formation [24].

Multiple mechanisms can explain the association between deeper or larger defects and infertility. These include pooling of menstrual blood within the niche, resulting in chronic inflammation; impaired contractility of the residual myometrium; alterations in the local uterine environment; and possibly an increased risk of chronic endometritis, as well as mechanical interference with embryo implantation or sperm transport [13]. The literature supports a higher prevalence of chronic endometritis in women with CSD (after matching for confounding factors), with odds ratios of ~1.5-1.8 [25]. These mechanisms help explain why imaging parameters, such as depth (and RMT), are not only morphological fossils but are also correlated with symptom severity and reproductive potential.

Given the frequent occurrence of AUB, pain, and infertility in women with CSD, clinical awareness should be raised in settings with high CS rates. Early diagnostic imaging, especially TVS supplementation, when needed with saline infusion sonohysterography or hysteroscopy [26], can be valuable [27,28]. Treatment options, including hysteroscopic resection [29] and laparoscopic/scar repair [30], have been shown to improve symptoms. For example, laparoscopic niche repair was reported to improve symptoms in approximately 77% of patients and to restore fertility in approximately 73% of patients in a recent observational cohort [23]. Clinicians should also consider morphological metrics (depth, residual myometrium) when counseling patients: deeper defects (> 3 mm in our study) are more likely to be associated with infertility and might benefit from more aggressive or earlier intervention.

The strengths of this study include a well-defined symptomatic cohort, the use of both imaging and confirmatory modalities, quantified measurement of defect depth, and correlational analysis with reproductive history. However, this study has several limitations. It is cross-sectional in design, with a relatively small sample size, and includes only symptomatic women without a control group, which may introduce selection bias. Associations were assessed using p-values only, without formal effect sizes, confidence intervals, or adjustment for multiple comparisons. Continuous variables were analyzed with t-tests, but normality testing was not formally performed. Potential confounders, such as BMI, parity, and cesarean indication, were not controlled for in the multivariate analysis. Imaging was primarily 2D TVS, and inter-observer reliability was not statistically quantified. Follow-up data on long-term reproductive outcomes were not collected, limiting conclusions regarding causality and the effectiveness of interventions. Despite these limitations, the study provides valuable insights into the clinical and imaging characteristics of CSD in reproductive-age women, which can inform future research and guide clinical awareness.

Future research should focus on prospective cohort studies tracking reproductive outcomes over time in women with different defect morphologies, including post-treatment outcomes. The incorporation of volume-based or 3D metrics may offer greater predictive power than simple depth or residual myometrium alone. In addition, randomized trials comparing different surgical repair techniques (hysteroscopic, laparoscopic, or vaginal) are needed to determine optimal management strategies. Finally, the development and validation of standardized diagnostic and classification criteria for CSD will improve the comparability across studies and guide treatment.

## Conclusions

CSD is an underrecognized but clinically significant consequence of cesarean delivery, associated with AUB, pelvic pain, and infertility in reproductive-age women. In this study, we identified key predictive associations between defect morphology and symptom severity, particularly that niche depth >3 mm and multiple prior CS were significantly linked with secondary infertility and irregular niche formation. These findings emphasize the need for early, imaging-based detection and clinical vigilance in women with a cesarean history who present with unexplained gynecological symptoms. Our study adds region-specific evidence from a low-resource setting, where standardized CSD assessment is rarely practiced, thereby contributing to the global understanding of how surgical and obstetric factors influence niche formation. Integrating CSD evaluation into the routine diagnostic workup for women with abnormal bleeding or infertility may improve reproductive outcomes. Future multicenter prospective studies with long-term follow-up are needed to establish standardized diagnostic criteria and assess the reproductive benefits of targeted surgical repair.
